# Diabetes and end-stage renal disease; a review article on new concepts

**DOI:** 10.12861/jrip.2015.07

**Published:** 2015-06-01

**Authors:** Seyed Bahman Ghaderian, Fatemeh Hayati, Shokouh Shayanpour, Seyed Seifollah Beladi Mousavi

**Affiliations:** ^1^Diabetes Research Center, Ahvaz Jundishapur University of Medical Sciences, Ahvaz, Iran; ^2^Chronic Renal Failure Research Center, Ahvaz Jundishapur University of Medical Sciences, Ahvaz, Iran

**Keywords:** Diabetic nephropathy, End-stage renal disease, Renal replacement therapy, Hemodialysis

## Abstract

It is well established that diabetic nephropathy is the most common cause or in combination with hypertensive nephropathy are the most common causes of end-stage renal disease (ESRD) in developed and developing countries. For this review, we used a variety of sources by searching through PubMed, Embase, Scopus, Current Content and Iran Medex from January 1990 up to December 2014. Manuscripts published in English and Persian languages, as full-text articles, and or as abstract were included in the study. Patient survival in diabetics on maintenance renal replacement therapy including hemodialysis (HD), peritoneal dialysis (PD) and kidney transplantation is significantly lower than that seen in nondiabetics with ESRD. The poor prognosis of diabetic patients with ESRD is partly due to presence of significant cardiovascular disease, problems with vascular access, more susceptible to infections, foot ulcer, and hemodynamic instability during HD. Although, many complications related to kidney transplantation may occur in diabetic ESRD patients, multiple studies have found that the kidney transplantation is the preferred renal replacement therapy for diabetic patients with ESRD and it is associated with a much better survival and quality of life than dialysis among these patients.

**Implication for health policy/practice/research/medical education:**

It is well established that diabetic nephropathy and hypertensive nephropathy are the leading cause of end-stage renal disease (ESRD) in developed and developing countries reflects the catastrophic squeals of these two silent killers. On the other hand, ESRD is a worldwide public health problem with an enormous financial burden for healthcare systems.

## Introduction

Diabetic nephropathy, classically defined by the presence of proteinuria occurs in significant percent of patients with type 1 which formerly called insulin-dependent and type 2 which formerly called non-insulin-dependent diabetes mellitus (DM). It also can occur in the patients with secondary forms of DM for example after pancreatitis or pancreatectomy if the duration of DM is longs-enough and level of glycemia high enough to result diabetic complications (1,2).

Approximately 20% to 30% of patients with type 1 DM will have microalbuminuria after a mean duration of diabetes of 15 years and less than half of these patients will progress to macroalbuminuria which also called overt nephropathy (3-5). After overt nephropathy development, the substantial number of patients will progress to end-stage renal disease (ESRD) with reported rates of 4% to 17% at 20 years and approximately 16% at 30 years from time of initial diagnosis of DM (1,2).

Although the prevalence of progressive renal disease generally lower estimated in type 2 diabetes, however, recent data suggest that the renal risk is currently equivalent and the time to proteinuria from the onset of diabetes and the time to ESRD from the onset of proteinuria were similar in the two types of diabetes (2,3).

A number of investigations have shown that the development and progression of diabetic nephropathy may be retarded by normalization of the blood pressure preferably with blockade of renin-angiotensin system (RAS) including angiotensin converting enzyme inhibitor (ACEI) and angiotensin II receptor blockers (ARB), use of other agent such as spironolactone an aldosterone antagonists, pentoxifylline a non-selective phosphodiesterase inhibitor and strict control of the plasma glucose concentration, however substantial number of patients still progress to ESRD (4-6). On the other hand, ESRD is a worldwide public health problem with an enormous financial burden for healthcare systems (7,8).

According to rapid increase in the prevalence of DM in general population and its complication, the aim of this review article is to evaluate the role of diabetes in cause of new ESRD patients requiring renal replacement therapy, survival of diabetic ESRD patients, comparison of hemodialysis (HD) versus peritoneal dialysis (PD) and comparison of dialysis versus transplantation among diabetic patients.

## Materials and Methods

For this review, we used a variety of sources by searching through PubMed, Embase, Scopus, Current Content and Iran Medex from January 1990 up to December 2014.The search was performed by using combinations of the following key words and or their equivalents: ‘end stage renal disease, ESRD, hemodialysis’, ‘dialysis’, ‘peritoneal dialysis’, ‘kidney transplantation’ in combination with ‘diabetic nephropathy. Manuscripts published in English and Persian languages, as full-text articles, and or as abstract were included in the study. Unfortunately we did not specifically hand search conference proceedings and manuscripts published in other languages.

## Diabetes as a cause of ESRD

Many studies were done about primary causes of ESRD in developed countries. In the past decades, several forms of glomerulonephritis (GN) were the most common initiating cause of ESRD in these countries. However because of more aggressive treatment of GN and possibly because of rapid increase in the prevalence of obesity and diabetes, it is well established that diabetic nephropathy is now the leading cause and or probably with hypertensive nephropathy are the most common causes of ESRD in developed countries. For example according to the result of United States renal data system, diabetes is the most common cause of ESRD, accounting for approximately 45% of cases (9-11).

An increasing incidence has also been noted in European countries, as reported, 34% and 30% of patients requiring renal replacement therapy have diabetes in Germany and Australia respectively (11,12). However, the incidence of patients who develop ESRD due to diabetes appears to have stabilized in Denmark, which may be due to the widespread implementation of intensive renoprotective measures such as improved glycemic and blood pressure control (12).

Although, there is lack of data about etiology of ESRD in developing countries, it appears that diabetes and hypertension are also the leading causes of ESRD similar to developed countries reflects the catastrophic sequelae of these two silent killers (13-16). For example, in Iran from 1997 to 2006, the percentage of new patients requiring renal replacement therapy, attributed to diabetic nephropathy increased two-fold from 16% in 1997 to 31% in 2006 (15).

In an other report from Iran, DM was the most common cause of ESRD accounting for approximately 35% of cases in patients aged 40 years and older*.* In addition, diabetic nephropathy and hypertensive nephrosclerosis together have 55% causative role in developing ESRD (13).

The results of other studies in developing countries also show that the cause of significant proportion of patients with ESRD is diabetic nephropathy (17-19). For example Al Wakeel et al (17) demonstrated that DM is the commonest cause of ESRD seen in 26.6% followed by nephrosclerosis in Saudi Arabia and it also was the most prevalent comorbidity seen during the study period and occurred in 59% followed by heart disease in 32.7% (17). High prevalence rates of DM have also been reported from other Arab countries including 21.2% in Kuwait, 35% in Egypt and 46.8% in Lebanon (18-20).

## Survival of diabetic patients with ESRD

Although maintenance dialysis prevents death from uremia and the life expectancy of patients with ESRD including diabetic patients has improved since the introduction of dialysis in the 1960s, it is still far below that of the general population. In addition, patient survival in diabetics on maintenance dialysis including HD and PD is lower than that seen in nondiabetics with ESRD in developed and developing countries (21-28).

The poor outcome of diabetic ESRD patients has demonstrated in many studies (24-26). As an example, according to the report of 2009 United States Renal Data System (USRDS), the 5 years survival of diabetes patients with ESRD is catastrophic and only 30% after initiation of HD. In comparison 1, 3, 5, and 10-year survival of ESRD patients on maintenance HD in the United States is 79%, 53%, 35%, and 11.2%, respectively. These values are also much far below than the general population (which the range of the expected remaining life span is 30 to 40 years for those aged 40 to 44, and 17 to 22 years for individuals aged 60 to 64) (27).

The result of Chantrel et al study also showed the poor survival of diabetic patients with ESRD in developed countries with relatively high survival rates. According to the result of this study which performed in a center in France, 32% of patients with type 2 diabetes requiring dialysis died after a mean follow-up of 211 days (27).

While many studies about the survival of diabetic ESRD patients on maintenance dialysis have been done in developed countries, however, there is few data from developing countries has been existed.

According to the two reports from Iran, the long-term survival among diabetic dialysis patients is catastrophic and lower than that seen in nondiabetic ESRD patients (22,25,27). In the first study, survival of 185 ESRD patients on intermittent HD was evaluated. Although, one-year survival of diabetic patients is approximately similar to nondiabetic patients, however, 3 and 5-year survival of diabetic patients is significantly lower (52.2% vs. 73.8%) and no one of diabetic patients had 5-year survival (0% vs. 56.9%) (22). In the other study, long-term survival of 1861 ESRD patients in multicenter of HD in southwest of Iran was investigated. The survival of diabetic patients was significantly lower than nondiabetic patients and 1, 5, 10 and 15-year survival of diabetic and nondiabetic patients are 79.2 vs. 85%, 32.3 vs. 11.5%, 5.8 vs. 0.4, and 1.7 vs. 0% respectively (27).

In summary, according to the results of above studies, the survival of diabetic dialysis patients is lower than nondiabetic patients. There is an important question now. Is there any difference between survivals of dialysis patients with DM as primary cause of ESRD with dialysis patients with DM as a comorbid condition?

There are few studies which compare this issue among dialysis patients. In a large international cohort study with using of data from the European Renal Association-European Dialysis and Transplant Association Registry, Schroijen et al (28) compare the survival of 15 419 dialysis patients; 3624 dialysis patients with diabetes as primary renal disease, 1193 dialysis patients with diabetes as a comorbid condition, and 10 602 dialysis patients without diabetes. According to the result of the study, the survival of both groups of diabetic dialysis patients was lower than nondiabetic patients. In addition, after adjusting for age, sex, country and malignancy, the survival of dialysis patients with diabetes as a primary renal disease is lower compared to patients with diabetes as a comorbid condition. Many factors contribute to poor prognosis of diabetic patients with ESRD, including presence of significant cardiovascular disease, problems with vascular access and lower lifetime of arteriovenous fistula, more susceptible to bacterial and fungal infections, foot ulcer and hemodynamic instability during HD due to autonomic neuropathy (20-28).

## Dialysis modality in diabetics

Choice of a dialysis modality including HD, chronic ambulatory peritoneal dialysis (CAPD) or automated peritoneal dialysis (APD) in diabetics is influenced by a number of considerations which apply to nondiabetics as well. Some of them include availability and convenience, comorbid conditions, socioeconomic and dialysis center factors, independence and motivation of the patient, ability to tolerate volume shifts, risk and history of infection, status of the peripheral vasculature to create adequate vascular access for HD and status of the abdomen for PD (29-39).

Diabetic patients particularly old age patients are more likely to have severe peripheral blood vessels disease that limits the ability to create and sustain adequate arteriovenous fistula for chronic HD. In addition, many of diabetic patients have autonomic neuropathy and therefore these patients are often more likely to have frequent hypotensive episodes during HD especially during ultrafiltration dialysis in which fluid removal is the primary goal. Development of intradialytic hypotension usually necessitates intravenous fluid replacement therapy and therefore volume overload among these patients. On the other hand, hypotensive episodes during HD, usually necessitates decrease of the blood flow rate and in some times, discontinuation of HD and therefore induces inadequate dialysis and some other significant complication among these patients (33-39).

In conclusion, diabetic patients can be treated with either PD or HD in a way that provides the advantages of each modality. However according to the above complication, it may be suggested that diabetic dialysis patients initially undergo PD and transfer to HD once complications ensue with PD. This is based in part on the hypothesis that compared with HD, PD may provide better preserve residual renal function and better short-term survival. However, analysis of data provided by the United States Renal Data Systems (USRDS) and other studies have found that survival benefit associated with PD is during the first few years on dialysis and is lost over time (30,34).

## Dialysis versus kidney transplantation

Many complications related to kidney transplantation may occur in diabetic ESRD patients. Compared with nondiabetic transplant recipients, the incidence of cardiovascular disease among diabetic patients is high and it cause highest rates of adverse cardiac events in the early and late posttransplant period. In addition, the risk of bacterial and fungal infections including posttransplant urinary tract infections is more common in diabetic versus nondiabetic transplant recipients. Another challenge in diabetic ESRD patients is glycemic control after transplantation. Because of immunosuppressive regimens used after transplantation and their detrimental effects on pancreatic beta cell function and peripheral insulin action, the glycemic control and achievement of target glucose levels are often more difficult after transplantation and therefore the prevention of recurrence of the diabetic lesions in the transplanted kidney is also difficult (40-44).

In contrast to above challenges and complications, several studies have found that kidney transplantation is the preferred renal replacement therapy for diabetic patients with ESRD. According to the results of these studies, kidney transplantation generally is associated with a much better survival and quality of life than dialysis among these patients (41,42). As an example, survival analysis using data from the USRDS have showed that, despite a significantly increased short-term mortality following surgery, the long-term survival is much higher among diabetic transplant recipients compared with patients on dialysis. Among 7200 diabetic transplant recipients, at 18 months after transplantation, the risk of death is reduced 73% compared with the approximately 15 000 diabetic placed on a waiting list for transplantation (relative risk: 0.27, 95% CI: 0.24-0.30). In addition, projected life expectancy among diabetic patients who underwent transplantation was 11 years compared with diabetics wait-listed patient (43).

The result of survival analysis from another study in Scotland among 1732 patients wait-listed for a first kidney transplant is also similar. At 12 months posttransplantation, the risk of death among approximately 250 diabetic patients who underwent transplantation was lower and the projected increase in life was significantly higher compared with diabetics who remain on dialysis (41).

It is suggested that, the reduction in mortality among dialysis patients who underwent kidney transplantation compared with patients who remain on dialysis due in part to a decrease in the risk of fatal and nonfatal cardiovascular complications especially among diabetic patients (44).

## Preemptive kidney transplantation

The current evidence suggests that all patients with chronic kidney disease (CKD) have a survival advantage with preemptive transplantation (before dialysis is required) when compared with initiation of dialysis followed by transplantation and preemptive kidney transplantation is recommended if possible rather than transplantation after a period of dialysis (45,46).

It seems that among diabetic patients with CKD, preemptive kidney transplantation rather than initiation of dialysis followed by transplantation is also preferred and it is also associated with substantial improvements in patient survival. As an example, according to the report of USRDS, relative to preemptive transplants, there is an increase in risk for death after transplantation of 21%, 28%, 41%, 53%, and 72% among 73, 103 patients including almost 20 000 diabetic patients with waiting times of 6 to 12 months, 12 to 24 months, 24 to 36, 36 to 48, and over 48 months, respectively. There is also an association between the risk of graft loss and increasing time on dialysis in all patients with ESRD, including patients with diabetes (45).

According to the results of current studies, it is not clear whether the better patient and allograft survival of preemptive transplantation among ESRD diabetic patients are achieved when either living donor or deceased donor kidneys are used. For example, the result of Meier-Kriesche et al (45) study showed that preemptive kidney transplantation is associated with better patient and allograft survival among both living and deceased donors. In contrast to the result of this study, Becker et al (46) in a retrospective study of over 20 000 diabetic patients have suggested that the benefit of preemptive transplantation is limited to living donor recipients. In this study, ESRD diabetic patients who underwent preemptive kidney transplantation from living donors have lower mortality (with relative risks of 0.57 and 0.65 for recipients with type 1 and type 2 diabetes, respectively) in contrast to either type 1 or type 2 diabetic recipients of cadaveric donor kidneys.

## Conclusion

Diabetic kidney disease occurs in the significant percentage of patients with type 1 and type 2 DM. Although the prevalence of progressive renal disease generally lower estimated in type 2 diabetes, however, recent data suggest that the renal risk is currently equivalent and the time to ESRD from the onset of proteinuria were similar in the two types of diabetes. It is well established that diabetic nephropathy particularly type 2 and hypertensive nephropathy are the leading cause of ESRD in developed and developing countries reflects the catastrophic sequelae of these two silent killers.

Patient survival in diabetics on maintenance dialysis including HD and PD is lower than that seen in nondiabetics with ESRD. It seems that, the survival of dialysis patients with diabetes as primary renal disease is also lower compared to patients with diabetes as a comorbid  condition.

Diabetic patients can be treated with either PD or HD. It may be suggested that diabetic dialysis patients initially undergo PD because of provide better preserve residual renal function and better short-term survival with PD. However, analysis of many studies have found that the survival benefit associated with PD is during the first few years on dialysis and is lost over time.

Although, many complications related to kidney transplantation may occur in diabetic ESRD patients, multiple studies have found that the kidney transplantation is the preferred renal replacement therapy for diabetic patients with ESRD and it is associated with a much better survival and quality of life than dialysis among these patients.

The current evidence suggests that diabetic patients with CKD similar to other CKD patients have a survival advantage with preemptive transplantation when compared with initiation of dialysis followed by transplantation and it is recommended if possible rather than transplantation after a period of dialysis. However, it is not clear whether the better patient and allograft survival of preemptive transplantation among ESRD diabetic patients are achieved when either living donor or deceased donor kidneys are used.

## Authors’ contribution

SBG and SSBM conducted the search and gathering the data. SS, HF and SBG prepared the primary draft. SSBM edited the manuscript.

## Conflicts of interest

The authors declared no competing interests.

## Ethical considerations

Ethical issues (including plagiarism, data fabrication, double publication) have been completely observed by the author.

## Funding/Support

None.
